# A label-free, fast and high-specificity technique for plant cell wall imaging and composition analysis

**DOI:** 10.1186/s13007-021-00730-9

**Published:** 2021-03-19

**Authors:** Huimin Xu, Yuanyuan Zhao, Yuanzhen Suo, Yayu Guo, Yi Man, Yanping Jing, Xinqiang He, Jinxing Lin

**Affiliations:** 1 College of Biological Sciences, China Agricultural University, Beijing, 100193 China; 2 Beijing Advanced Innovation Center for Tree Breeding by Molecular Design, Beijing Forestry University, Beijing, 10083 China; 3 College of Biological Sciences and Biotechnology, Beijing Forestry University, Beijing, 100083 China; 4 Institute of Tree Development and Genome Editing, Beijing Forestry University, Beijing, 100083 China; 5 School of Life Sciences, Peking University, Beijing, 100871 China; 6 Biomedical Pioneering Innovation Center, Peking University, Beijing, 100871 China

**Keywords:** Coherent Raman scattering, Coherent anti-Stokes Raman scattering, Stimulated Raman scattering, Cell wall, Label-free imaging, Chemical composition

## Abstract

**Background:**

New cell wall imaging tools permit direct visualization of the molecular architecture of cell walls and provide detailed chemical information on wall polymers, which will aid efforts to use these polymers in multiple applications; however, detailed imaging and quantification of the native composition and architecture in the cell wall remains challenging.

**Results:**

Here, we describe a label-free imaging technology, coherent Raman scattering (CRS) microscopy, including coherent anti-Stokes Raman scattering (CARS) microscopy and stimulated Raman scattering (SRS) microscopy, which can be used to visualize the major structures and chemical composition of plant cell walls. We outline the major steps of the procedure, including sample preparation, setting the mapping parameters, analysis of spectral data, and image generation. Applying this rapid approach will help researchers understand the highly heterogeneous structures and organization of plant cell walls.

**Conclusions:**

This method can potentially be incorporated into label-free microanalyses of plant cell wall chemical composition based on the in situ vibrations of molecules.

## Background

Plant cell walls are a renewable biomaterial composed of lignin and various polysaccharides in a complex three-dimensional matrix. The chemical composition of plant cell walls varies not only between tissues and cell types but also along developmental gradients and even within a single cell wall [1–3]. To understand this variation, major efforts are currently exploring cell wall composition and structure in the native state, mostly by applying high-resolution approaches at the scale of single cells or cell wall layers.

Over the past two decades, various plant cell wall imaging techniques have been developed to analyze plant cell wall microstructure and chemical composition [4–9], such as bright- and dark-field microscopy [10], polarized light microscopy [11], transmission electron microscopy (TEM) [12], scanning electron microscopy (SEM) [12]. Moreover, histochemical and immunolabeling techniques have been applied to investigate the dynamics of different plant wall polysaccharides [9, 13, 14]. Visualization of plant cell wall lignification using fluorescence-tagged monolignols has also been reported [15–17]. Furthermore, genetically encoded fusion proteins (combining a marker protein with a fluorescent protein) have become useful for labeling cell walls, enabling relatively simple visualization of the cell walls of living plants. In addition, fluorescently labeled samples of plants have been imaged by light-sheet fluorescence methods, such as selective-plane illumination microscopy (SPIM) [18]. This labeling technique served as a basis for the development of confocal laser scanning microscopy (CLSM) and stimulated emission depletion microscopy imaging systems, which have significantly improved the temporal-spatial resolution and signal-to-noise ratio of cell wall imaging [19, 20].

Rapid advances in imaging technology are improving our understanding of the spatial heterogeneity of individual cell walls, as well as taxonomic-level differences between cell walls [5]. For example, new, label-free imaging techniques have evolved into powerful tools to detect and characterize the complex chemical and structural composition of the plant cell wall. Compositional information can be obtained through a variety of label-free imaging technologies, such as atomic force microscopy (AFM) [21–23], Fourier-transform infrared (FTIR) spectroscopy [7, 24], and confocal Raman microscopy (CRM) [25–27]. However, these methods have disadvantages such as the cumbersome and time-consuming sample preparation, serious damage to samples, the generally non-quantitative data output, making it difficult to follow the dynamic processes involved in plant development or the conversion of biomass to other forms (such as saccharification) at high spatiotemporal resolution [19].

Here, we present coherent Raman scattering (CRS) microscopy, including coherent anti-Stokes Raman scattering (CARS) microscopy and stimulated Raman scattering (SRS) microscopy, as an effective method for imaging the complex chemistry of plant cell walls based on molecular vibrations in biological molecules. This method is free from interference due to sample autofluorescence, can observe substances that are difficult to label, and is not dependent on exogenous labels of cellular components such as lignin and large biomolecules. CARS microscopy offers a dramatic improvement in signal levels over spontaneous Raman microscopy, thus offering a significantly reduced scanning rate at several seconds per frame (512 × 512 pixels) and making video-rate imaging possible [28]. In addition, epi-detected CARS (E-CARS) microscopy with two synchronized picosecond pulse trains was reported to greatly improve the image contrast via effective rejection of the solvent background [29, 30]. SRS technology overcomes the limitations of previous techniques, allows the use of vibrational contrast [31], offers a signal that is orders of magnitude stronger than spontaneous Raman microscopy, and exhibits straightforward image interpretation and quantification without interference from the non-resonant background and phase-matching conditions [32]. Because SRS is free of non-resonant background, its signal intensity is linearly dependent on the analyte concentration only [33]. These merits enable background-free, in situ quantitative microanalysis of cellular structure and chemical composition in live plant tissues [34, 35]. Moreover, high-speed imaging makes it easy to capture a dynamic process in whole living cells with a diffraction-limited spatial resolution of ~ 350 nm and a pixel dwell time of 50 μs [36].

Below we describe the necessary steps and typical obstacles encountered when attempting to quantify the chemical composition in plant cell walls. Then, we discuss the process we used in our previously published work in more detail. This protocol will allow the interested reader to address fundamental questions regarding the chemical and structural composition of plant cell walls, with applications in basic research, biorefineries, and the pulp industry.

## Materials

### Reagents

This protocol does not require specific reagents. Distilled water and ethyl alcohol are sufficient for sample preparation.

### Equipment

- Sliding microtome (Leica SM2010 R)
- CRS microscope. The integrated CARS and SRS imaging system is a custom-built system with a commercial laser source (APE PicoEmerald, Germany) and an optimized inverted microscope (Olympus FV1000, Japan). The picosecond laser emits synchronized pump and Stokes beams. The pump beam is tunable from 700 to 990 nm and consists of an 80-MHz pulse train with a 2-ps pulse width. The 1031-nm Stokes beam is modulated by a built-in electro-optic modulator (EOM) at 20 MHz. The pump and Stokes beams are overlapped and coupled into an inverted laser-scanning microscope optimized for near-infrared throughput. Non-descanned detectors are used for CARS imaging. A detector and a lock-in amplifier (customized from APE, Germany) are used for SRS imaging. This imaging system can perform CARS and SRS imaging simultaneously.

### Software

- Fiji is an open-source image processing package based on ImageJ, which bundles together many plugins that facilitate scientific image analysis (https://imagej.net/Fiji).
- The FV10-ASW3.0 software is used to control the microscope (https://www.photonics.com/Product.aspx?PRID=47380).

### Plant samples

#### Protocol

Our protocol consists of three main steps: sample preparation, acquisition of spectra, and computational analysis. Moreover, computational analysis can be semi-quantitative or fully quantitative.

#### Sample preparation

Tissues of herbaceous and woody plants can be used for CRS imaging. We recommend using at least three biological replicates, although more might be necessary depending on the question being addressed. If possible, use fresh samples or samples that have been kept at 4 ℃, or − 80 ℃. Dried material is not suitable. The micro cuttings could be obtained with conventional microtechniques, allowing for better data quality by generating sections of equal thickness, which helps to produce sharp images and substantially reduces the risk of reporting inaccurate differences between samples. In our work, a sliding microtome (option a) was used to prepare woody and solid samples. Although no defined thickness is required for the analysis, it is crucial to have a planar surface with intact cell walls, as the inelastic scattering, not the absorption, is measured. Importantly, the interference from chloroplast fluorescence can be severe in some samples. Therefore, for many plant samples, appropriate embedding and chlorophyll removal is key.

### Acquisition of spectra: CRS imaging settings

**Fig. 1:**
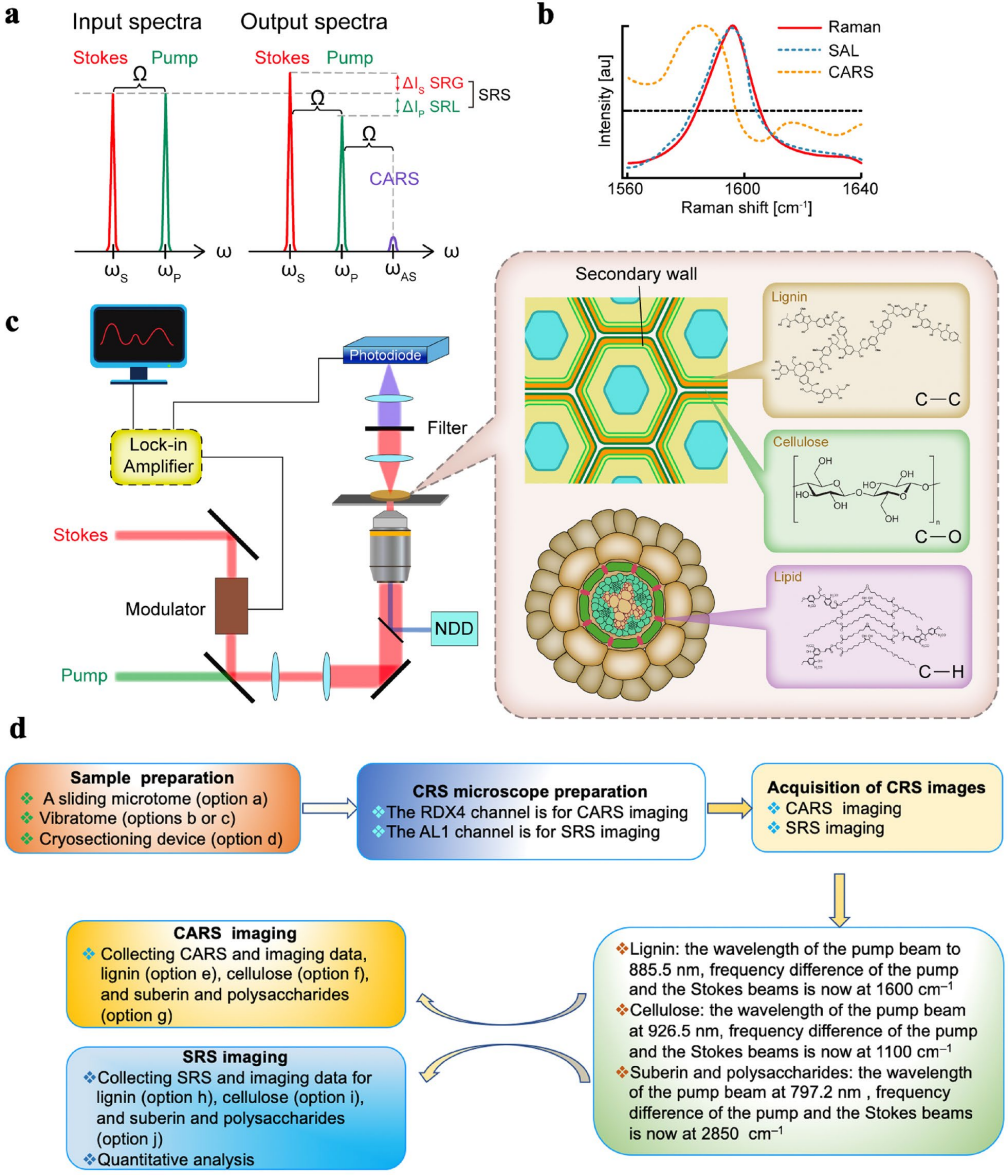
Principle and design of CRS microscopy. Modified from Freudiger et al. [33]. CRS microscopy mainly includes two sub-types, coherent anti-Stokes Raman scattering (CARS) microscopy and stimulated Raman scattering (SRS) microscopy, both of which can be performed by setting the wavelength of the pump beam in a single setup. **a** The principle of SRS microscopy. Two input beams (Stokes and pump) are focused on the sample; when the difference in energy between the two beams (Ω) matches that of a specific chemical bond in the sample, then an additional signal is produced. Input and output spectra of SRS and CARS is shown. SRS leads to an intensity increase in the Stokes beam (SRG) and an intensity decrease in the pump beam (SRL). Also shown (not to scale) is the CARS signal generated at the anti-Stokes frequency ω_AS_ when the energy difference between the pump beam photon and the Stokes beam photon matches the vibrational frequency (Ω_vib_) of a specific chemical bond. **b** Agreement of the SRL spectrum (red circles) with the spontaneous Raman spectrum (black line) of the Raman peak (1595 cm^−1^) of 10 mM retinol in ethanol. The distorted CARS spectrum (blue squares) exhibits a typical peak shift, a dispersive shape, and non-resonant background. **c** Plant cell wall imaging of the chemical composition of stem and root tissues. A CRS microscope with forward and epi detection is illustrated. The Stokes beam is modulated by an electro-optic modulator. The transmitted or reflected pump beam is filtered and detected by a large-area photodiode. The SRL is measured by a lock-in amplifier to provide a pixel of the image. The CARS signal is detected by the NDD. **d** Schematic of the process of CRS imaging of plant cell wall composition

The laser intensity should be sufficiently high to obtain good counts per molecule (CPM) and to ensure a good signal-to-noise ratio, but also sufficiently low to avoid photobleaching, photodamage, and saturation effects during the CRS measurement. The principle and design of CRS microscopy are shown in Fig. 1. In SRS, two laser beams at ω_p_ and ω_S_ coincide on the sample (Fig. 1a). When the difference in frequency, Δω = ω_p_ _−_ ω_s_, also called the Raman shift, matches a particular molecular vibrational frequency ω, amplification of the Raman signal is achieved by virtue of stimulated excitation. Consequently, the intensity of the Stokes beam, I_s_, experiences a gain, ΔI_s_ (stimulated Raman). Unlike SRL and SRG, CARS exhibits a non-resonant background (Fig. 1b). CARS microscopy achieves far stronger Raman signals, but suffers from a non-resonant electronic background that can limit the sensitivity for low-concentration species. Figure 1c shows the experimental scheme used to image the plant cell wall, together with the chemical composition of the stem and roots. The experimental scheme of CRS imaging of plant cell wall composition is shown in Fig. 1d.

### Quantitative analysis of SRS-calibrated images

This section comprehensively describes the guidelines for image and data processing. The calibrated images allow an estimate of the protein distribution in specific cellular compartments, organelles, or structures. For that purpose, an additional fluorescent marker is used. Intensity analyses for specific types of cell walls were performed with Fiji software. The program then selected pixels with intensity values above the set threshold for statistical analysis. For each type of plant sample, no less than three images were selected for intensity analysis. Lignin (1600 cm^−1^) signals in each pixel were plotted. We recommend meticulously following the critical steps of the procedure, and researchers can optimize the analyses according to their own needs.

### Preparation of the plant samples by a sliding microtome (Leica SM2010 R)

Time: 30 min–1 h.

**Fig. 2:**
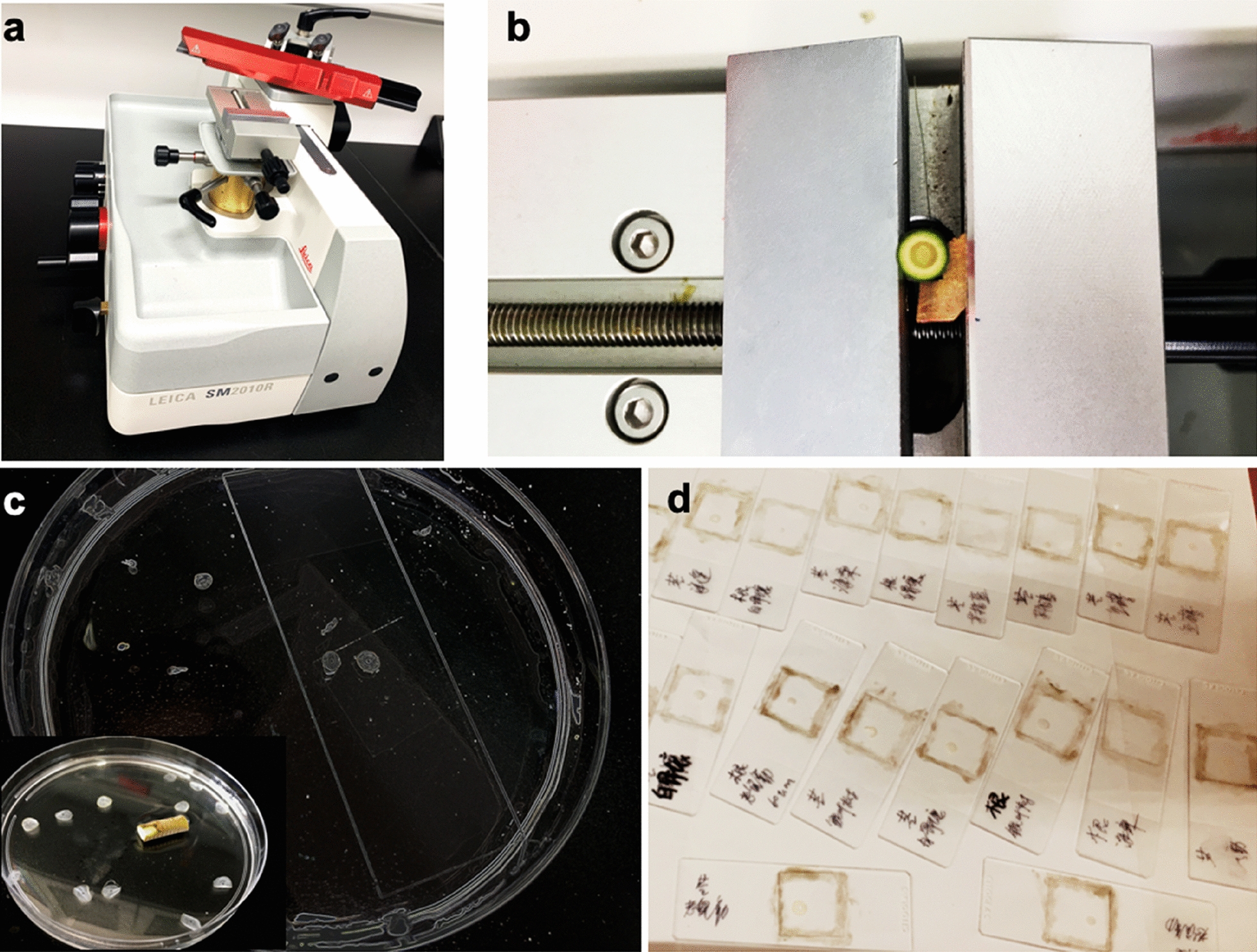
Micro cuttings can be obtained with conventional techniques. **a** Sliding microtome. **b** The plant samples were cut into 10-μm-thick slices, and these cross-sections were gently washed using distilled water, then were transferred from the buffer tray into a glass beaker and then to a clear Petri plate (**c**). **d** The sections were transferred to the slide, with a coverslip and sealed

1. Clamp the specimen in the universal cassette clamp.
2. Replace the pressure plate; both the pressure plate and the matching insertion aid must be replaced.
3. To replace them, push the knife guard toward the right and push the lever upward to release the clamp of the pressure plate. Carefully pull the insertion aid out and to the left. The pressure plate can now be taken off.
4. To mount another pressure plate, proceed in the reverse order. Only use the pressure plate together with the matching insertion aid.
5. To loosen the clamp, rotate the eccentric lever upward, and orient the specimen in the cutting direction by turning the setscrews.
  *Note: Turning the lever further to the left re-clamps the orientation.*
6. When clamping a disposable blade, handle the microtome knives or blades carefully. The blade holder must be installed in the instrument before a blade is inserted.
7. Inserting the knife: lock the knife sledge in place using the locking knob. Please make sure that the knife holder is firmly clamped using the clamping lever and that the knurled head screw is tightened. Push the knife guard to the right and loosen the clamping screws sufficiently to allow the knife to be inserted. Take the knife out of the knife case and insert it carefully. Tighten the two clamping screws in alternation until both are secure, and cover the knife with the knife guard.
8. Cutting the specimen (trimming). Adjust the section thickness setting, and then hold the knife sledge at the grip and place the sledge behind the specimen.
9. Pull the knife guard of the blade/knife holder to the right. To feed the specimen towards the knife, turn the coarse drive wheel.
10. Select the required section thickness (10 µm) with the section thickness adjusting knob and move the manual feed lever. Each lever movement causes a specimen to feed by the selected amount.
11. Move the knife sledge back and forth until the specimen surface is trimmed, as required.
  *Note: The blades are extremely sharp; careful attention is required.*
12. Collect the sections using a disposable plastic pipette during sectioning in the buffer tray. Alternatively, the sections can be transferred from the buffer tray into a glass beaker and then to a clear Petri plate.
13. Then transfer the sections to the slide and seal the slide for CRS imaging (Fig. 2). Spread the section on the slide with a fine brush and add one drop of fresh H_2_O. Pick up a coverslip (cleaned with Kimwipes) with a tweezer and carefully place it on top. Seal the corners of the coverslips to keep them from falling off when placed inversely on the microscope stage.
  *Note: Avoid air bubbles in the liquid film.*

### Acquisition of CRS images

Plant cell wall samples should be sectioned into slices, placed on glass slides, and sealed as described above. Spread the section on the slide with a fine brush and add one drop of fresh H_2_O. Pick up a coverslip (cleaned with Kimwipes) with the help of a tweezer and carefully place it on top. Stick the corners of the coverslip to keep them from falling off when placed inversely on the microscope stage. Adjust the *X*–*Y* axes of the microscope stage to place the sample directly above the objective lens. Adjust the *Z* axis so that the sample is in the focal plane in the bright-field mode. CRS and imaging data can now be collected.

*Note: The working distance of the objective lens is very short. Adjust the Z axis very carefully to avoid contact between the objective lens and the coverslip.*

### Collecting CARS and imaging data for lignin (option e), cellulose (option f), and lipids (option g) of the cell wall as examples

Time: 0.1 h of setup, 0.2 h of measurement.

**Fig. 3:**
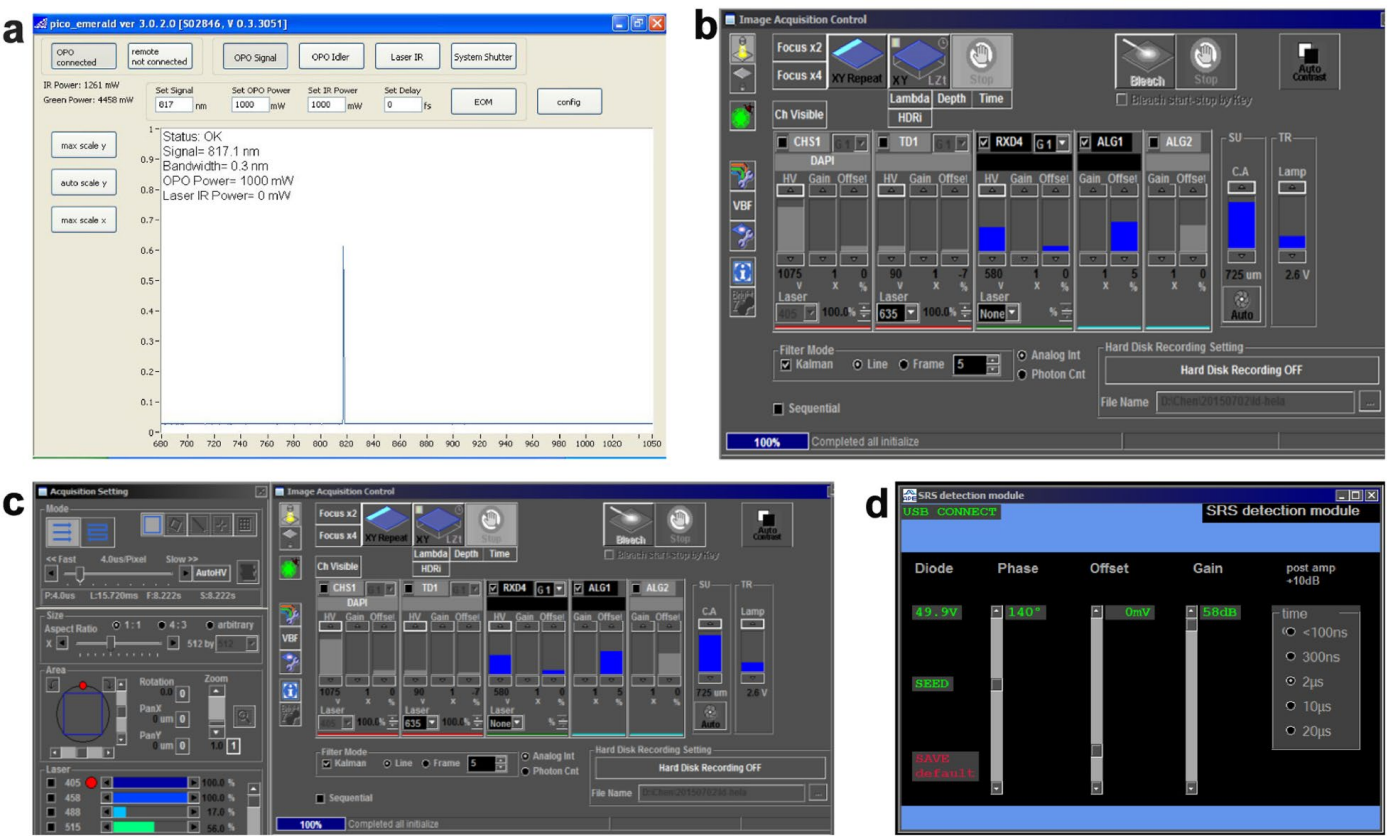
Detection and acquisition of CRS images. **a** Control software setting. Turn on the key switch and power switch of the laser control unit. Then turn on the power switch of the chiller and check whether the water-cooled pipe joint is leaking. Set the temperature to 23 ℃. Wait about 0.5 h for the temperature to stabilize. **b** Dialog window for detection channel settings. Switch on the panel PC. The control software (picoEmerald ver 3.0.2.0) will start automatically (**a**). Confirm by clicking “YES” when the system asks you whether to start the laser. This takes about 0.5 h to warm up and light the laser. **c** Dialog window for image settings. Turn on the main power switch, key switch, scanning module, and motorized translation stage of the microscope. **d** Dialog window for SRS detection settings. Open the FV10-ASW3.0 software of the microscope by double-clicking the FV10-ASW3.0 icon. The RDX4 channel is for CARS imaging, while the AL1 channel is for SRS imaging

(e) Collecting CARS images of lignin

1. Set the wavelength of the pump beam to 885.5 nm by ticking the “Set Signal” box. The frequency difference of the pump and the Stokes beams is now at 1600 cm^−1^, matching the aromatic ring vibration frequency of lignin.
2. Set the power of the pump and Stokes beams by ticking the “Set OPO Power” and the “Set IR Power” boxes, respectively. It is safe to set the power to 200 mW for each beam in this example.
  *Note: If the laser power is too high, the samples might be burned.*
3. Toggle the “OPO Signal”, “Laser IR”, and “System Shutter” buttons to let the pump and Stokes beams enter the microscope.
4. Move the stage and optimize the focus to identify an area containing the xylem cells of wood samples or herbaceous samples in the bright-field mode.
5. Set the scanning speed to 4.0 μs per pixel. Set the image size as 512 × 512 pixels. Check the RXD4 checkbox to activate the CARS channel. Deactivate other channels.
6. Begin scanning by clicking the “XY Repeat” button (Fig. 3). Finely adjust the *X*, *Y*, and *Z* axes to focus on the area of interest.
  *Note: If there is no signal, increase the laser power and adjust the voltage of the NDD detector by changing the value of “HV” on the RXD4 channel. Make sure there are no overexposed pixels. The best laser power and “HV” value will generate the best contrast of CARS images.*
7. Check the “Kalman” checkbox in the “Filter Mode”. Type in “5” for the line average. Click the “XY” button to start CARS imaging. Save the image to your desired file path.
8. Background measurement. Toggle the “Laser IR” button in the laser control software to block the Stokes beam. Click the “XY” button to start CARS imaging. Save the image to a file path.
9. Repeat Steps (e) 2–8 to image different views.
10. Repeat Steps (e) 2–9 for different samples.

(f) Collecting CARS images of cellulose

1. Set the wavelength of the pump beam to 926.5 nm by ticking the “Set Signal” box. The frequency difference of the pump and the Stokes beams is now at 1100 cm^−1^, matching the C–O stretching vibration frequency of the polysaccharides.
2. Repeat Steps (e) (2–8) for imaging of different views.
3. Repeat Steps (f) (1–2) for different samples.

(g) Collecting CARS images of lipids

Set the wavelength of the pump beam to 797.2 nm by ticking the “Set Signal” box. The frequency difference of the pump and the tokes beams is now at 2850 cm^−1^, matching the C–H stretching vibration frequency of the lipids.

1. Repeat Steps (e) (2–8) for imaging of different views.
2. Repeat Steps (g) (1–3) for different samples.

### Collecting SRS imaging data for lignin (option h), cellulose (option i), and lipids (option j) of the cell wall as examples

Time: 0.1 h of setup, 0.2 h of measurement.

(h) Collecting SRS images of lignin

1. Set the wavelength of the pump beam to 885.5 nm by ticking the “Set Signal” box. The frequency difference of the pump and the Stokes beams is now at 1600 cm^−1^, matching the aromatic ring vibration frequency of lignin.
2. Activate the built-in electro-optic modulator by toggling the “EOM” button in the “EOM Control” software.
3. Set the power of the pump and Stokes beams by ticking the “Set OPO Power” and “Set IR Power” boxes, respectively. It is safe to set the power to 200 mW for each beam in this example.
  *Note: If the laser power is too high, the samples might be burned.*
4. Toggle the “OPO Signal”, “Laser IR”, and “System Shutter” buttons to let the pump and Stokes beams enter the microscope.
5. Open the control software of the SRS detection module (Fig. 3). Set the integral time as 2 μs.
  *Note: The “SEED” indicator should be green if the lock-in amplifier of the detection module is connected correctly.*
6. Move the stage and optimize the focus to find an area containing the xylem cells of wood samples or herbaceous samples in the bright-field mode.
7. Set the scanning speed to 4.0–16 μs per pixel. Set the image size as 512 × 512 pixels. Check the AL1 checkbox to activate the SRS channel. Deactivate other channels.
8. Begin scanning by clicking the “XY Repeat” button. Finely adjust the *X*, *Y*, and *Z* axes to focus on the area of interest.
  *Note: If there is no signal, increase the laser power and adjust the “Phase” and “Gain” in the control software of the detection module to achieve the best contrast of SRS images.*
9. Check the “Kalman” checkbox in the “Filter Mode”. Type in “5” for the line average. Click the “XY” button to start SRS imaging. Save the image to a file path.
  *Note: Do not move the microscope stage.*
10. Repeat Steps (h) (6–9) for imaging of different views.
11. Repeat Steps (h) (6–10) for different samples.

(i) Collecting SRS images of cellulose

1. Set the wavelength of the pump beam to 926.5 nm by ticking the “Set Signal” box. The frequency difference of the pump and the Stokes beams is now at 1100 cm^−1^, matching the C–O stretching vibration frequency of the polysaccharides.
2. Repeat Steps (h) (2–9) for imaging of different views.
3. Repeat Steps (i) (1–2) for different samples.

(j) Collecting SRS images of lipids/suberin

1. Set the wavelength of the pump beam to 797.2 nm by ticking the “Set Signal” box. The frequency difference of the pump and the Stokes beams is now at 2850 cm^−1^, matching the C–H stretching vibration frequency of the lipids.
2. Repeat Steps (h) (2–9) for imaging of different views.
3. Repeat Steps (j) (1–2) for different samples.

### Quantitative analysis using SRS images

Time: 10 min of setup, 0.5–1 h of unattended computation (depending on the number of measurements), 30 min of quality control, and 0.5–1 h of image processing.

1. Add folders to the Fiji path (using ‘File/Set Path/Add Folder’ from the Fiji menu). New empty folders are used to save the output results.
2. Intensity analyses for specific types of cell walls can be performed in Fiji software. Open the selected picture and change the image to 8 bit (Fig. 6a).
3. Click on “Analyze” and select “Histogram” to calculate the average photon counts for all measurements (Fig. 6b).
4. Click on “List” to view the intensity values and counts (Fig. 6c, d).
5. The total intensity values will be divided into 256 parts. From the 0 to 255 value, the intensity counts will be calculated for every tenth value. Then the ratio of the intensity value will be obtained by dividing the sum of ten counts by the total counts.
6. For each type of plant sample, at least three images should be selected for intensity analysis. Lignin (1600 cm^−1^) signals in each pixel are plotted as intensity histograms and normalized by total intensity for better comparison.

### Potential experimental pitfalls

1. Poor resolution of the CRS spectrum. This may occur if the sections of the sample are too thick, or if the slit is too wide. Therefore, make sure the sections are ≤ 10 μm thick and that the slit opening is ≥ 50 μm.
2. Too much background fluorescence or other fluorescence interference. To prevent this, be sure to use new coverslips and prepare a fresh sample for imaging, ensure the samples are not damaged, and ensure there are no bubbles. Additionally, the sections of the samples can be immersed in 75% ethanol before spreading onto the slide to avoid chloroplast auto-fluorescence.
3. Sample burning. The sections of the samples were embedded in LR White resin, the samples are too thin, or the laser intensity is too high. Both herbaceous and woody samples should be prepared from fresh but not embedded. Also, the laser intensity should be sufficiently high to obtain good counts per molecule (CPM) and to ensure a good signal-to-noise ratio, but also sufficiently low to avoid photobleaching.

## Results and discussion

### Sample preparation

Various plant cell wall imaging methods have been developed and have revealed novel details of the cell wall structure and chemical composition, as well as providing information that has proven valuable in the exploitation of biomass polymers [16, 37, 38]. Sectioning of the stem tissue is the first step for visualizing cell walls by these techniques, which poses some challenges: (i) cumbersome sample preparation and difficulty of sample isolation when small cell wall areas or single cell layers are of interest, since they have to be carefully excised; (ii) damage to samples caused by chemical treatment and photobleaching during fluorescence observation [38]. For example, FTIR spectroscopy is perturbed by the strong infrared absorption of water and, thus, requires the samples to be dried [39].

Except vibratome, cryo-sectioning microtome [7, 40], the micro cuttings could be obtained with other microtomes. In our work, various fresh plant materials were sectioned via conventional microtechniques by a sliding microtome (Fig. 2), allowing us to achieve better data quality by generating sections of equal thickness, which helps to produce sharp images and substantially reduces the risk of reporting inaccurate differences between samples. A suitable microtome should be selected based on the characteristics of the sample, and the sections of the sample can be immersed in 75% ethanol before spreading onto the slide to remove chlorophyll (Table 1). Compared to embedding specimens in resin, this method has the advantage of keeping the tissues alive and reducing sample manipulation, as well as saving time.

**Table 1:** The problems and solution methods during the protocol

Number	Problem	Possible reason	Solution
1	A lack of good contiguous microsections	An inappropriate microtome was used	A suitable microtome should be selected based on the characteristics of the sample
2	Intensity is too low	Laser intensity is too low	Make sure the intensity of the laser at the sample position is correct; increase laser power accordingly
		Exposure time is insufficient	Increase the exposure time
		Samples are overtreated and not suitable for imaging	Prepare the samples carefully according to step 1
3	Poor spectral resolution of the CRS spectrum	The sections of the sample are too thick, or the slit is too wide	Make sure the sections are ≤ 10 μm thick and that the slit opening is ≥ 50 μm
4	Too much fluorescence of background	Coverslips are dirty, there are bubbles, or the samples are damaged	Use new coverslips or prepare a new sample for imaging
5	Other fluorescence interference	Chloroplast auto-fluorescence	The sections of the sample can be immersed in 75% ethanol before spreading onto the slide
6	Sample burns	The sections of the sample were embedded in LR White resin or laser intensity is too high	Both herbaceous and woody samples should be prepared from fresh or dry plant material but not embedded The laser intensity should be sufficiently high to obtain good counts per molecule (CPM), to ensure a good signal-to-noise ratio, but also sufficiently low to avoid photobleaching

### CRS imaging of cell walls at the macroscopic scale

Cell wall composition is particularly important in plants grown for forage, biofuels, and biomaterials (i.e., wood for construction). In our previous study, we investigated the cytological characteristics and chemical composition of the lipid portion of suberin in endodermal cells during the process of Casparian strip formation using SRS imaging in maize (*Zea mays*) primary roots, suggesting that SRS microscopy is a promising, reliable, and quantitative method to perform noninvasive, label-free imaging of Casparian strips in vivo [41]. CARS has also been used to gauge the chemical and structural composition of cell walls of birch (*Betula platyphylla*), oak (*Quercus palustris*), and spruce (*Picea asperata*) samples [42].

**Fig. 4:**
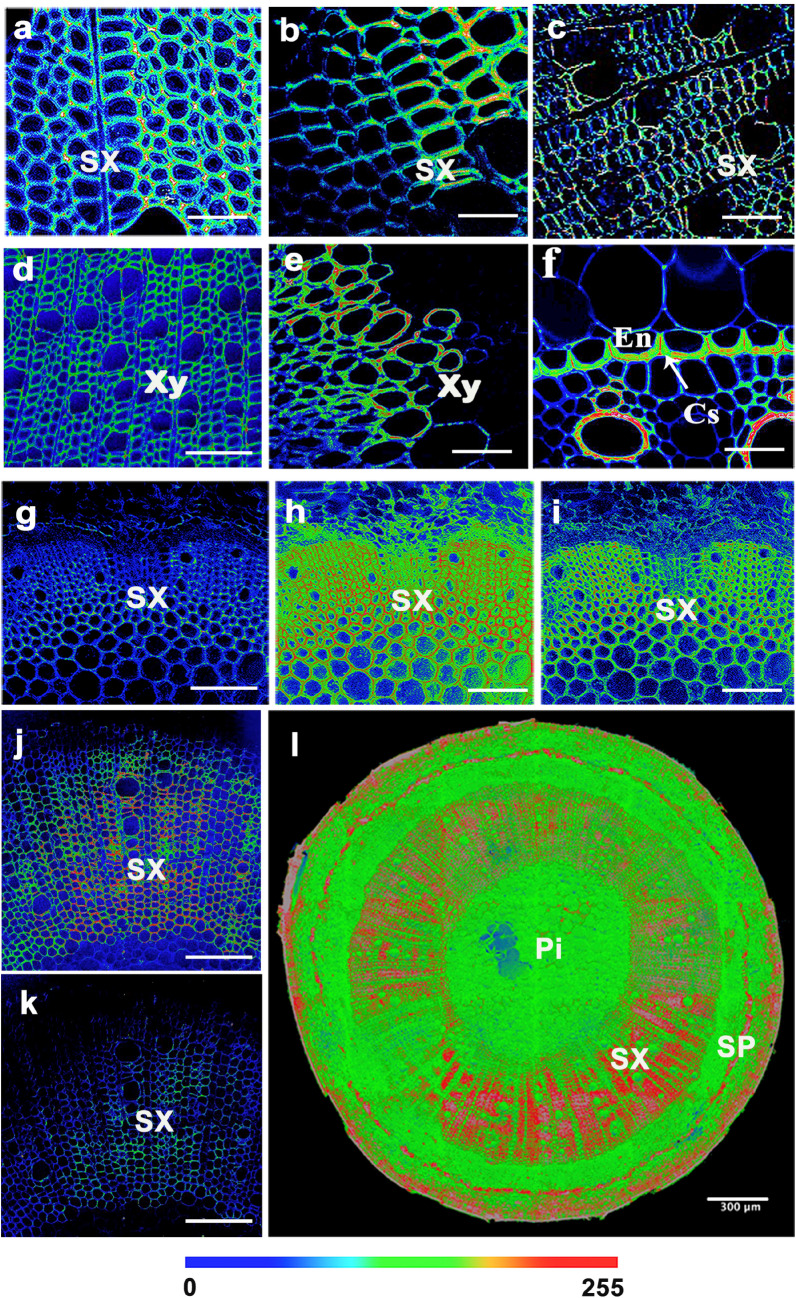
CRS images of lignin, cellulose, and lipids in xylem cell walls. **a**–**c** CARS images of lignin, cellulose, and lipids in poplar, respectively. **d**, **e** SRS images of lignin of stem xylem in poplar and cellulose of stem xylem in Arabidopsis. **f** SRS image of the cell walls in the Casparian strip of a maize root. **g**–**i** SRS images of lignin, cellulose, and lipids in the secondary xylem cell wall of *Bruguiera sexangula* stem tissue, respectively. **j**–**l** SRS images of lignin, cellulose, and lipids in the secondary xylem cell walls of *Derris trifoliata*. *SX* secondary xylem, *Xy* xylem, *Cs* Casparian strip, *En* endodermis, *SP* secondary phloem, *Pi* pith. Scale bars = 50 µm in **a**–**k**, 300 µm in **l**

Here, to further collect CARS and SRS images of cell walls, we followed the experimental schematic shown in Fig. 1d and set the appropriate wavelength of the pump beam and the frequency difference of the pump and Stokes beams depending on the biomolecules of interest (Fig. 2). The cross-sections were observed using CRS microscopy and images were acquired at 1600 cm^−1^ (C–C), 1100 cm^−1^ (C–O), and 2805 cm^−1^ (C–H) for lignin, cellulose, and lipids, respectively (Fig. 3). CARS images of lignin, cellulose, and lipids in *Populus* are shown in Fig. 4a–c. SRS images of lignin in the xylem cell walls of *Arabidopsis thaliana* root and stem tissues are shown in Fig. 3d, e.

**Fig. 5:**
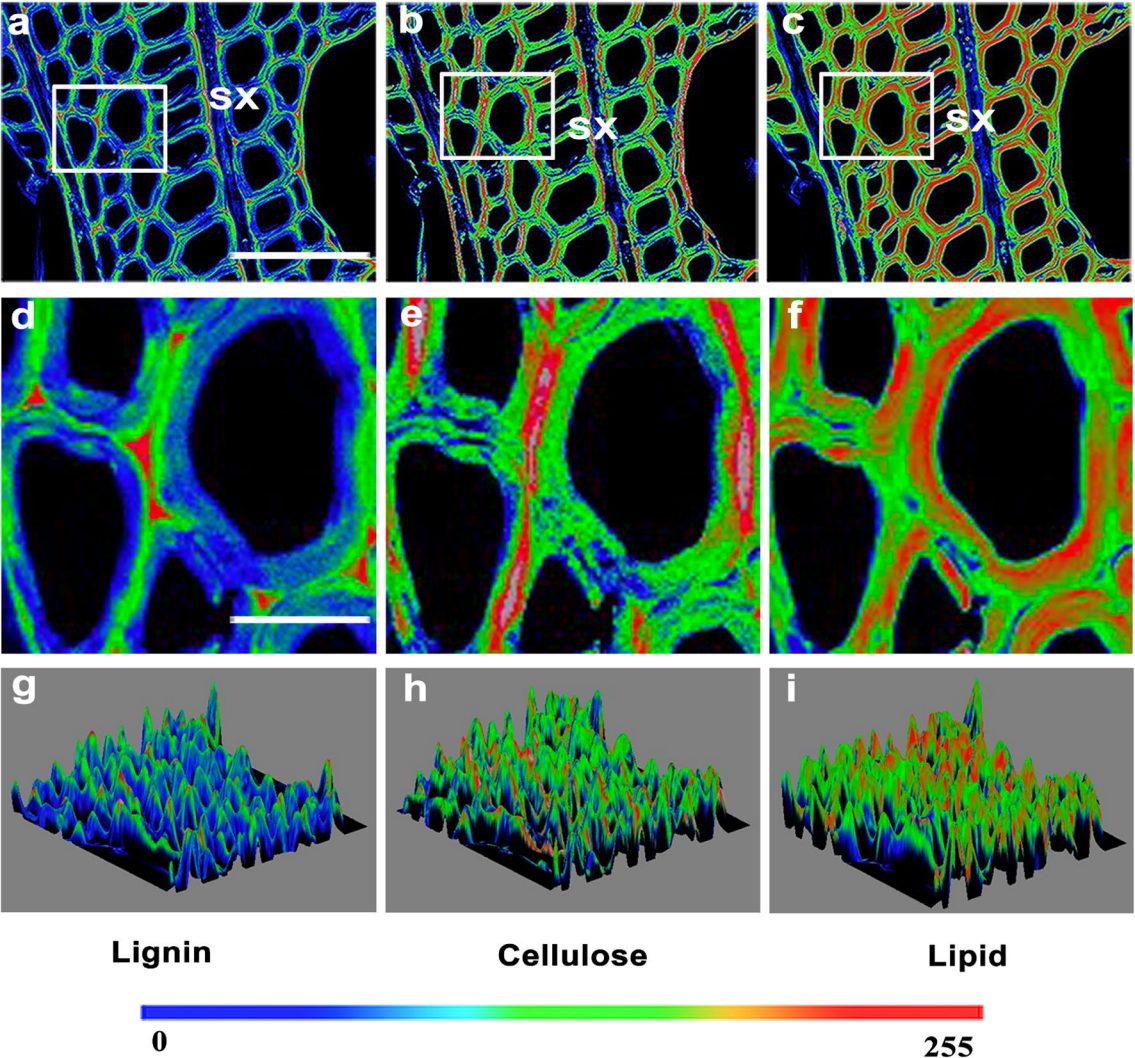
SRS images of lignin, cellulose, and lipids in xylem cell walls. **a**–**c** SRS images of lignin, cellulose, and lipids in the secondary xylem cell walls of poplar, respectively. The white boxes are magnified in **d**–**f**. The 3D surface plots are shown in **g**–**i**. *SX* secondary xylem; Scale bars = 50 µm in **a**–**f**

Casparian strips form a network in the primary walls of endodermal cells. Here, we also imaged lignin and lipids of suberin in the Casparian strips of maize roots by SRS microscopy, showing the development of Casparian strips (Fig. 4f). The cytological characteristics and lignin, cellulose, and lipids of cell walls in stems of the salt-tolerant plant *Bruguiera sexangula* were also investigated (Fig. 4g–l). In addition to these images of specific areas, a whole-stem cross section SRS image of *B. sexangula* is shown in Fig. 4l. Interestingly, the higher-magnification and 3D surface plots of poplar cell wall SRS images (Fig. 5) showed that in large vessel cells, lignin appears to be mainly deposited in the spaces between adjacent cells (Fig. 5d), cellulose was evenly distributed in primary cell walls and the middle lamella (Fig. 5e), and the lipid signals were mainly observed in primary cell walls (Fig. 5f). Some troubleshooting was needed in our examples, such as ensuring the intensity of the laser at the sample position was correct, and that the laser power was increased accordingly (Table 1).

The ability to characterize in situ molecular vibrations has created exciting new opportunities for cell wall imaging. Notably, compared to traditional Raman methods, CRS has been used to analyze substances that are difficult to label, such as polysaccharides and large biomolecules, including lignin, achieving higher orders of resolution and better sensitivity. This protocol offers researchers a more comprehensive tool using simplified methods with label-free, fast, and high-specificity imaging.

This protocol uses CRS approaches, including CARS and SRS, for imaging complex biomolecules based on their molecular vibrations. CRS microscopy has been extensively applied to detect the distribution of and quantify cell wall components [19, 41–43]. Although CARS microscopy offers label-free chemical imaging with high sensitivity and high spatial resolution, its signal also suffers from a non-resonant electronic background component that can, to a certain degree, distort the chemical information of interest, making quantitative image interpretation challenging. In addition, background signal detection is still a technical problem in SRS, and considerable engineering efforts are needed to resolve this challenge. Other long-term goals in the further optimization of unlabeled imaging technologies include how to further distinguish different chemical components, especially how to better use the Raman peak molecular characteristics to observe the dynamic distribution and behavior of specific molecules in living cells.

### SRS computational analysis of cell wall composition

Understanding the multiscale structures of cell wall will contribute to reveal their function in plant growth and development, and accurate analytical techniques are broadly used in cell wall research [44]. SRS is another label-free imaging method and a promising technique for imaging chemical bonds based on the photo-switching concept of stimulated emission depletion [45, 46]. In our previous study, we examined the effects of the microRNA miR857, which is involved in the regulation of lignin content and consequently the morphogenesis of secondary xylem. The intensity of the lignin signals among wild-type, lignin-deficient lines (overexpressing *35S:MIR857*), and lignin-overproducing (*mir857* mutant) plants in Arabidopsis were analyzed by SRS microscopy [43]. In addition, compared with images stained using the traditional chemical dye FY 088, SRS images at the CH_2_ stretching vibration show a higher sensitivity and specificity for the lipid component of suberin lamellae, especially with respect to the latent capacity of SRS images to acquire a signal for a low-abundance substance in the primary site of suberin without a non-resonant background [41]. Additionally, besides used for the for plant lignification imaging [47], we also reported that this protocol worked well for samples from seeds of *Jatropha carcas* L., an emerging oil crop. The oil bodies in endosperm cells were imaged at 2850 cm^−1^, highlighting oil bodies rich in CH_2_ at 24 and 48 h after *Jatropha carcas* L. seed inoculation with sterile water [48].

**Fig. 6:**
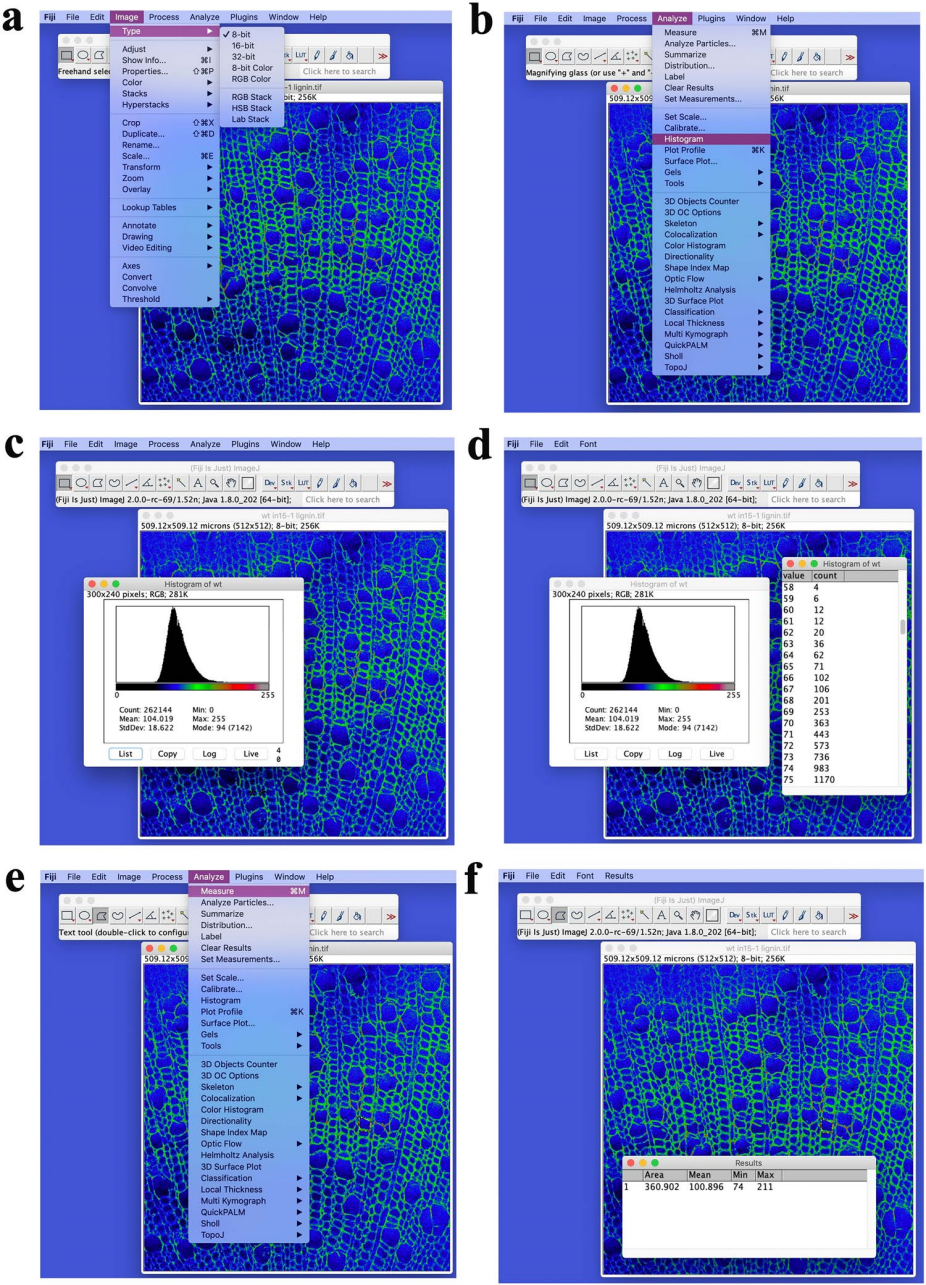
Quantitative analysis of SRS images of lignin in secondary xylem cell walls in poplar. **a** Image settings. Intensity analyses for specific types of cell walls can be performed in Fiji software. Open the selected picture and change the image to 8 bit. **b** Calculate the average photon counts for all measurements. Click on “Analyze” and select “Histogram” to calculate the average photon counts for all measurements. **c**, **d** Obtain the ratio of the intensity value. Click on “List” to view the intensity values and counts. The total intensity values will be divided into 256 parts. From the 0 to 255 value, the intensity counts will be calculated for every tenth value. Then the ratio of the intensity value will be obtained by dividing the sum of ten counts by the total counts. **e**, **f** Calculate the average intensity for selected cells. For each type of plant sample, at least three images were selected for intensity analysis. About 50 cells were selected for further analysis. Click on “Analyze” and select “Measure” to calculate the average intensity for selected cells. Lignin (1600 cm^−1^) signals in each pixel are plotted

**Fig. 7:**
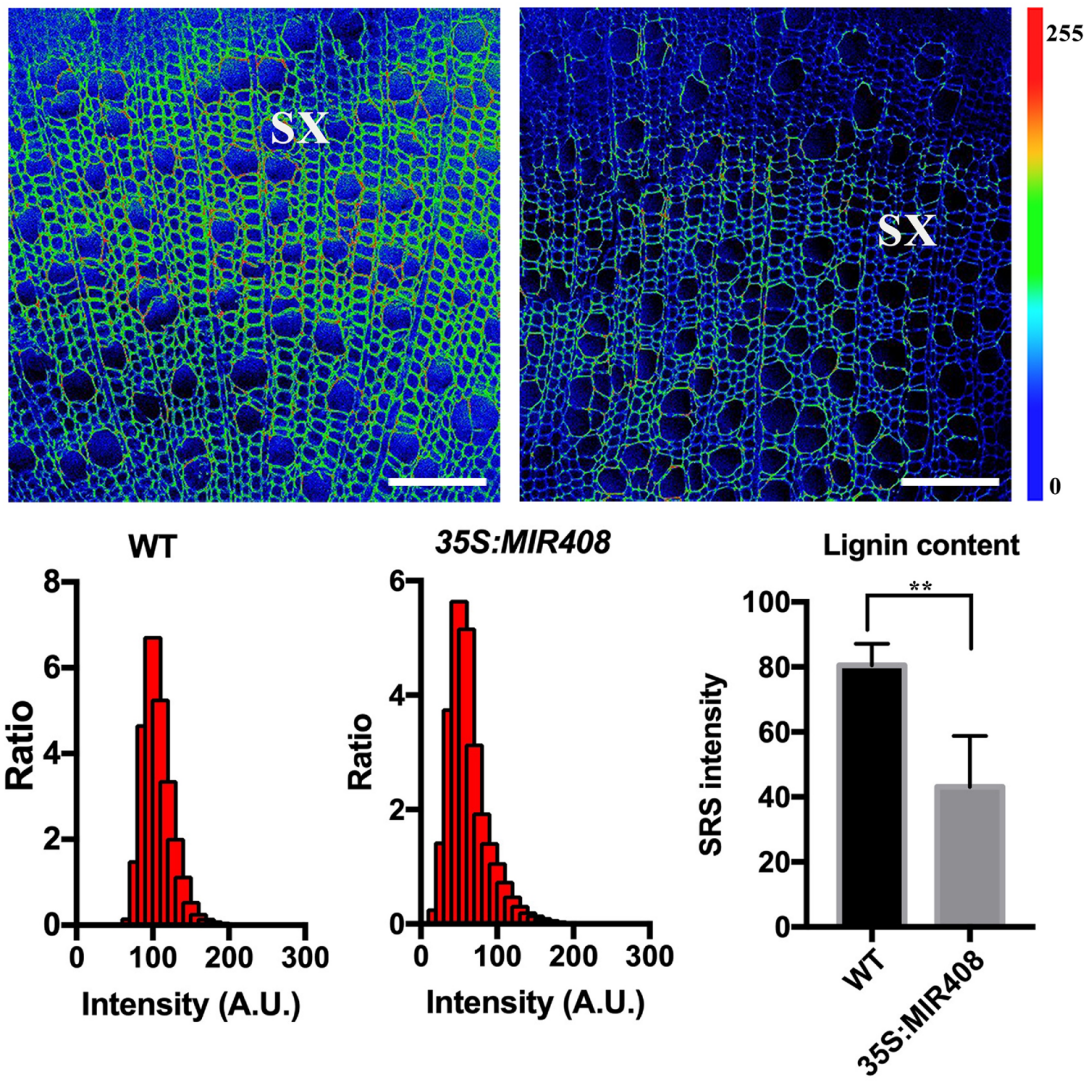
Example of quantification of lignin in stems using SRS images. SRS images of wild-type (WT) and *35S:MIR408*-overexpressing poplar plants acquired at 1600 cm^−1^, showing the lignin distribution. Color code indicates an increase of the signal intensity from blue to red. Scale bars = 50 µm

To further analyze the distribution of chemical components in cell walls in woody plants, we used quantitative and straightforward SRS image analysis, as shown in Fig. 6. In our example, we analyzed the intensity of the lignin signals among wild-type and lignin-deficient lines (overexpressing *35S:MIR408*), and then used image analysis software, such as Fiji or ImageJ, to calculate the fluorescence intensity of the cell wall in the secondary xylem. Individual cells were manually selected from the SRS image acquisition, in combination with further image analysis steps. Fluorescence intensity analysis of specific types of cell wall samples can be computed as described in the methods. The fluorescence intensity of the cell wall in the secondary xylem is lower and more narrowly distributed in poplar plants overexpressing *35S:MIR408* than in the wild type (Fig. 7), as well as the lignin content.

Although chemical imaging of poplar wood cell walls was conducted by CRM [7, 25], the quantitative analysis of this data was challenging. Compared to CRM, SRS microscopy is free of non-resonant background and linearly dependent on the analyte concentration, thus offering quantitative and straightforward image analysis [35]. These results confirmed that our CRS images of the cell wall can be used for further analysis of chemical composition, which offers a superior alternative for fast, large-area biological chemical imaging in living, intact cells.

## Conclusions

In summary, the cell wall imaging techniques described here can detect specific chemical bonds within particular molecules, which is applicable to real-time in situ quantification in plant tissues with high chemical specificity and spatial resolution. Therefore, CRS can overcome the limitations of existing techniques and offers the possibility to uncover remarkable new insights into the mechanisms of plant biology by nondestructively gathering spatially resolved chemical information and enabling quantitative microanalysis of the chemical composition of plant cell walls, which could be widely used in plant cell research, the pulp industry, and biofuel analysis.

## Data Availability

All data generated or analyzed during this study are included in this published article.
